# A case report of Tubo-ovarian abscess caused by *Burkholderia pseudomallei*

**DOI:** 10.1186/s12879-018-2986-z

**Published:** 2018-02-08

**Authors:** Pattaranit Nernsai, Areepan Sophonsritsuk, Srithean Lertvikool, Artit Jinawath, Maria Nina Chitasombat

**Affiliations:** 1 Division of Infectious Disease, Department of Medicine, Faculty of Medicine Ramathibodi Hospital, Mahidol University, 270 Rama VI Road, Ratchathewi, Bangkok, 10400 Thailand; 2 Department of Obstetrics and Gynecology, Faculty of Medicine Ramathibodi Hospital, Mahidol University, Bangkok, Thailand; 3 Department of Pathology, Faculty of Medicine Ramathibodi Hospital, Mahidol University, 270 Rama VI Road, Ratchathewi District, Bangkok, Thailand

**Keywords:** *Burkholderia pseudomallei*, Melioidosis, Ovarian abscess, Pelvic inflammatory disease

## Abstract

**Background:**

Melioidosis, the disease caused by *Burkholderia pseudomallei* is endemic in the Northeastern part of Thailand, South-East Asia, and Northern Australia. The pelvic involvement of disease is rare even in an endemic area. Therefore, we describe in this report the clinical presentation, management, and outcome of the patient with primary tubo-ovarian abscess due to melioidosis.

**Case presentation:**

A 31-year-old Thai cassava farmer woman presented with fever and abdominal pain at left lower quadrant for one month. She also had pain, swelling, and redness of the genitalia without any ulcer. She had odorless whitish vaginal discharge. The pelvic examination revealed excitation pain on the left side of her cervix. Transvaginal ultrasonography revealed a large left tubo-ovarian abscess size 9.4 × 4.8 cm located at anterior of the uterus. Urgent exploratory laparotomy revealed left hydrosalpinx with a large amount of pus. The pus culture grew *Burkholderia pseudomallei*. The computer tomography of the abdomen revealed multiple hepatosplenic abscesses. The patient underwent left salpingo-oophorectomy and pus drainage. The pathological examination of excised left adnexa revealed chronic and acute suppurative inflammation with necrotic tissue. She was given intravenous ceftazidime for one month, and her clinical symptom improved. She was diagnosed with type 2 diabetes mellitus at this visit and treated with insulin injection. She continued to take oral co-trimoxazole for 20 weeks. The final diagnosis was disseminated melioidosis with left tubo-ovarian abscess and hepatosplenic abscesses in a newly diagnosed morbidly obese diabetic patient.

**Conclusion:**

*Burkholderia pseudomallei* should be considered as the causative organism of gynecologic infection among patient with risk factor resided in an endemic area who do not respond to standard antibiotics. The pus culture from the site of infection is the only diagnostic method of pelvic melioidosis, appropriate antibiotics, and adequate surgical drainage were the components of the successful outcome.

**Electronic supplementary material:**

The online version of this article (10.1186/s12879-018-2986-z) contains supplementary material, which is available to authorized users.

## Background

Melioidosis, the disease caused by *Burkholderia pseudomallei* is endemic in the Northeastern part of Thailand, South-East Asia, and Northern Australia [1–3]. The organisms is a facultative intracellular aerobic gram-negative bacteria found in the environment mostly soil and surface water [4]. The possible routes of acquisition are direct cutaneous inoculation, inhalation, and ingestion [4]. Predisposing factors for melioidosis are diabetes mellitus, renal insufficiency, thalassemia, alcoholism and chronic lung disease [5]. The most common clinical presentation includes pneumonia, hepatic or splenic abscess, skin and soft tissue infection, urinary tract infection and osteoarticular infection [6, 7]. Gynecologic involvement is rare even in an endemic area, only a few brief report in literature. Therefore, we describe in this report the clinical presentation, management, and outcome of the patient with primary tubo-ovarian abscess due to melioidosis.

## Case presentation

A 31-year-old Thai cassava farmer woman from Prachinburi, a province in the Eastern region of Thailand, presented to a University Teaching Hospital in Bangkok with fever and abdominal pain for one month (see Additional file 1: Figure S1). Her gynecologic history was notable for three caesarean section and a tubal ligation. She had regular sexual intercourse with her husband. She had no other medical condition. She developed constant dull aching severe abdominal pain at left lower quadrant area over one month. She also had persistence high-grade fever without chills. She complained of pain, swelling, and redness of the genitalia without any ulcer. She also had a moderate amount of odorless whitish vaginal discharge. She lost eight kilograms within one month unintentionally. She visited the gynecologist and received two weeks prescription of oral doxycycline and metronidazole. Her swollen genitalia resolved; however, she still had abdominal pain, vaginal discharge, and high-grade fever. As a cassava farmer, she used to sit on the ground in the field and swim in the pond.

On presentation, the patient’s body mass index was 48 kg/m2. Her vital signs were as follows: body temperature, 39.5 °C; blood pressure, 120/60 mmHg; pulse, 98 beats/min; respiratory rate, 20 breaths/min. On physical examination, the skin, respiratory and cardiovascular system were clinically normal. No lymphadenopathy. Abdominal exam revealed transverse low abdominal surgical scar, mild distended, normoactive bowel sound, moderate tenderness at left lower quadrant area, no rebound tenderness, no guarding, no palpated mass palpable. Liver and spleen were not palpable. Splenic dullness was negative. The genital exam revealed mild erythema of labia majora, no ulcer. The pelvic examination revealed mild erythema external genitalia, minimal brownish discharge within vagina and excitation pain on the left side of her cervix.

Initial laboratory result. The complete blood count revealed the white blood cell count 15,300 cells/mm^3^ with 79% neutrophil. Hematocrit of 28%, hemoglobin concentration of 8.8 g/dL, platelet count of 531,000 cells/mm^3^, BUN 6 mg/dL, Cr 0.55 mg/dL, fasting glucose of 305 mg/dL, HbA1C of 13.94%. Liver function test showed AST 33 U/L, ALT 13 U/L, ALP 266 U/L, TB 0.5 mg/dL, DB 0.3 mg/dL, alb 18.7 g/L, glob 39.0 g/L (Table 1).

**Table 1:** Laboratory data on admission

Parameter	Recorded value	Standard value
White blood cell count	15,300 cells/mm^3^	4500–7500 cells/mm^3^
Neutrophils	79%	
Hemoglobin	8.8 g/dL	11.3–15.2 g/dL
Hematocrit	28%	36–45%
Platelet count	531,000 cells/mm^3^	130,000–350,000 cells/mm^3^
Total protein	57.7 g/L	69–84 g/L
Albumin	18.7 g/L	39–51 g/L
Total bilirubin	0.5 mg/dL	0.2–1.2 mg/dL
Direct billirubin	0.3 mg/dL	0.1–0.3 mg/dL
Aspartate aminotransferase	33 U/L	11–30 U/L
Alanine aminotransferase	13 U/L	4–30 U/L
Alkaline phosphatase	266 U/L	44–147 U/L
Blood urea nitrogen	6 mg/dL	8–20 mg/dL
Creatinine	0.55 mg/dL	0.63–1.03 mg/dL
Fasting glucose	305 mg/dL	70–109 mg/dL
Hemoglobin A1c	13.94%	< 6.5%

**Fig. 1:**
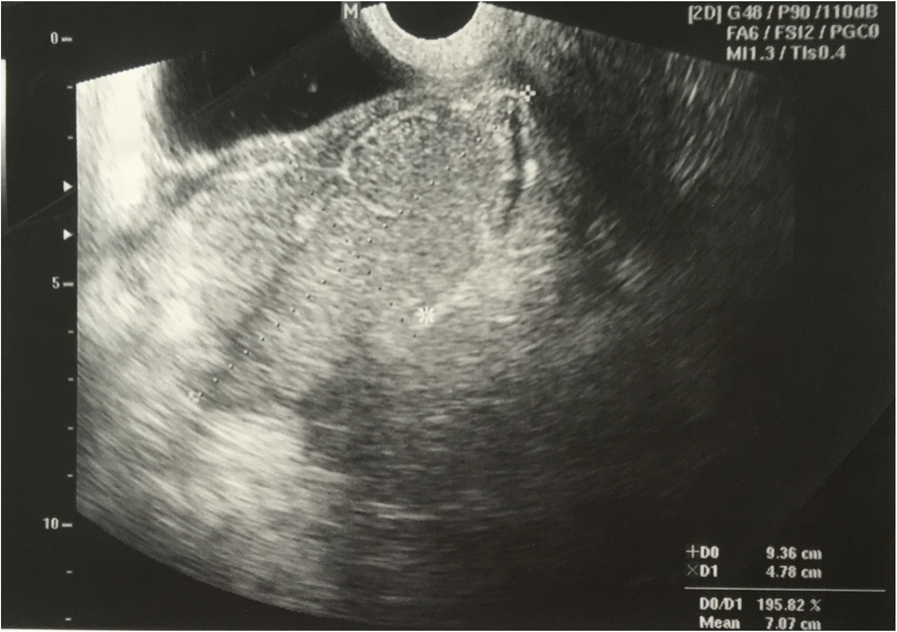
Transvaginal ultrasonography demonstrating a large left tubo-ovarian abscess size 9.4 × 4.8 cm located at anterior of the uterus

**Fig. 2:**
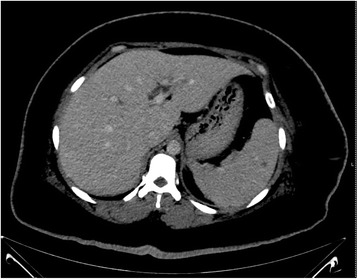
Computer tomography of whole abdomen demonstrating multiple small-size splenic abscesses

Her chest X-ray was normal. She was diagnosed with type 2 diabetes mellitus and had glycemic control with insulin injection. Transvaginal ultrasonography revealed a large left tubo-ovarian abscess size 9.4 × 4.8 cm located at anterior of her uterus (Fig. 1). The hemoculture revealed no growth of the organism. She received gentamicin and clindamycin intravenously empirically for nine days; however, she did not improve. Urgent exploratory laparotomy revealed left hydrosalpinx with pus collection amount 100 ml located between anterior wall of the uterus and left fallopian tube extended to anterior rectus sheath and rectus muscle. The pus was drained and sent for bacterial culture, and antibiotic sensitivity testing investigation. Initial culture results showed a scanty growth of *Burkholderia pseudomallei*. Treatment then commenced according to the sensitivity pattern on day 14 of admission. Serum antibodies to melioid antigen using an in-house indirect hemagglutination (IHA) test were positive at a titre of 1:2560. Further investigation with computer tomography of the whole abdomen revealed multiple splenic abscesses measuring 0.5–1.3 cm in size and a 0.8 cm liver abscess (Fig. 2). On day 17 of admission she developed surgical wound dehiscence and underwent the second exploratory laparotomy. Operative findings revealed left tubo-ovarian abscess size 4 × 5 cm adhered to left pelvic wall with pus loculated between left rectus sheath and muscle amount of 20 ml.

**Fig. 3:**
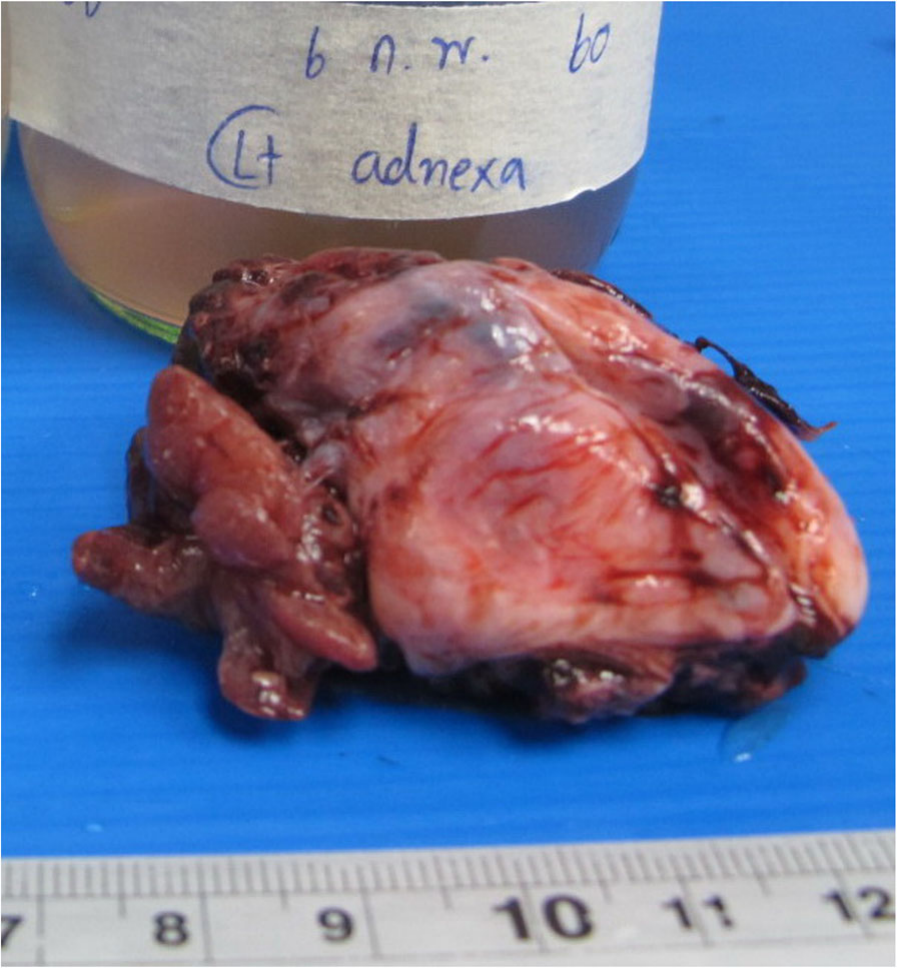
Gross appearance of the left adnexa demonstrating tumor-like lesion coated with old blood and fibrinous material

**Fig. 4:**
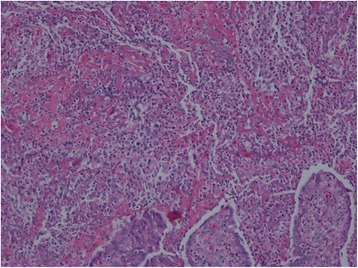
Histopathology of left fallopian tube and ovary. Power photomicrograph: [200× Hematoxylin and eosin stain] revealed chronic and acute suppurative inflammation with necrotic tissue

The left salpingo-oophorectomy and pus drainage were done (Fig. 3). The pathological examination of excised left adnexa revealed chronic and acute suppurative inflammation with necrotic tissue (Fig. 4). After 4 weeks of intravenous ceftazidime, her clinical symptom was improved and repeated ultrasonography of whole abdomen revealed resolution of pelvic collections and hepatosplenic abscesses. She was discharged from the hospital and continue oral co-trimoxazole for 20 weeks.

The final diagnosis was disseminated melioidosis with left tubo-ovarian abscess and hepatosplenic abscesses in newly diagnosed diabetic patient.

## Discussion

We presented a case of tubo-ovarian abscess due to *B.pseudomallei* in a diabetic patient. Tubo-ovarian abscess due to *B.pseudomallei* is extremely rare even in the endemic area [6–9]. We postulated that the patient had primary infection of genital tract by *B.pseudomallei* resulted from cutaneous inoculation of external genitalia by mean of exposure to soil and caused tubo-ovarian abscess by means of ascending infection. Later on, the abscesses extended to the abdominal wall and spread to liver and spleen during the time course period of inappropriate antibiotics. Surgical drainage of abscesses was crucial in term of both treatment and diagnosis. While initial vaginal swab culture grew *Lactobacillus* spp. and blood cultures were negative; only cultures from abscesses grew *B. pseudomallei.*

The pelvic involvement is extremely rare even in an endemic region of Thailand; a recent large prospective study of 626 patients with melioidosis, reported only two cases (cervicitis and tubo-ovarian abscess) [9]. The summary of gynecologic manifestation of melioidosis from literature included genital ulcer, cervicitis, pelvic inflammatory disease, tubo-ovarian abscess [10–12]. Complications included hepatic and splenic abscesses [10, 11]. Gestational infection leads to catastrophic outcome; fetal abortion and neonatal sepsis [12, 13]. Gestational infection in maternal could present with severe cystitis, vaginal discharge and an indolent ulcer or transient fever without positive blood culture [12, 13]. Microbiological diagnosis was made from urine and cervical discharge culture which grew *B. pseudomallei* and another case only cervical culture grew *B. pseudomallei* [12, 13]. Among all cases, blood cultures were negative, the microbiological diagnosis was revealed from pus culture from the site of infection; cervix, urine or abscess [12, 13].

## Conclusion

*Burkholderia pseudomallei* should be considered as the causative organism of gynecologic infection among patient with risk factor resided in an endemic area who do not respond to standard antibiotics. The pus culture from the site of infection is the only diagnostic method of gynecologic melioidosis, appropriate antibiotics, and adequate surgical drainage were the components of the successful outcome.

## Additional file

Additional file 1: Timeline. Patient clinical course. (DOCX 54 kb)

## Abbreviations

alb: albumin
ALP: Alkaline phosphatase
ALT: Alanine aminotransferase
AST: Aspartate aminotransferase
BUN: Blood urea nitrogen
Cr: Creatinine
DB: Direct bilirubin
glob: globulin
TB: Total bilirubin
