# Supplementary material for: A case report of Tubo-ovarian abscess caused by Burkholderia pseudomallei
Source: BMC Infect Dis. 2018 Feb 8;18:73. doi: 10.1186/s12879-018-2986-z (PMC5806353; doi:10.1186/s12879-018-2986-z)
Supplement: Supplementary file 1 — Timeline. Patient clinical course. (DOCX 54 kb) [file 12879_2018_2986_MOESM1_ESM.docx]

Clinical resolution

Exploratory laparotomy

Finding: left hydrosalpinx with pus loculated between anterior wall of the uterus and left fallopian tube extended to abdominal wall

Procedure: pus drainage

Symptoms did not improve:

high grade fever with abdominal pain

Second exploratory laparotomy: Finding: left tubo-ovarian abscess size 4 x 5 cm adhered to left pelvic wall with pus loculated between left rectus sheath and muscle

Procedure: left salpingo-oophorectomy with pus drainage

Pus culture: *Burkholderia pseudomallei*

Diagnosis: Melioidosis tubo-ovarian abscess

CT of whole abdomen: multiple splenic abscesses measuring 0.5-1.3 cm in size and a 0.8 cm liver abscess

Diagnosis: Disseminated melioidosis with left tubo-ovarian abscess and hepatosplenic abscesses

Consult for infectious disease medicine: changed antibiotic to ceftazidime IV 2g q 8hrs for 4 weeks

Surgical wound dehiscence

Diagnosis: Left tubo-ovarian abscess

Transvaginal ultrasound: 9.4x4.8 cm abscess at left adnexa

Diagnosis: Pelvic inflammatory disease

Outpatient Gynecologist evaluation for fever and abdominal pain at left lower quadrant with vaginal discharge, swollen of genitalia for one month

Pelvic exam: brownish discharge from vagina, pelvic excitation pain positive on left side

Ultrasound of whole abdomen:

resolution of intraabdominal abscesses

February 6, 2017

31-year-old Thai woman, Cassava farmer, Newly diagnosed DM type 2

Oral Doxycycline plus Metronidazole for 2 weeks

Discharged from hospital with oral TMP/SMX

Complete resolution of symptom on following up at 20 wks

IV Clindamycin plus gentamicin IV

Admitted to OB-GYN ward

Followed up at OPD OB-GYN:

symptoms did not improve

January 29, 2017

January 20, 2017

March 5, 2017

February 1, 2017

January 6, 2017

February 28, 2017
